# Transcriptomic and Epigenomic Landscape in Rett Syndrome

**DOI:** 10.3390/biom11070967

**Published:** 2021-06-30

**Authors:** Domenico Marano, Salvatore Fioriniello, Maurizio D’Esposito, Floriana Della Ragione

**Affiliations:** Institute of Genetics and Biophysics ‘A. Buzzati-Traverso’, CNR, 80131 Naples, Italy; domenico.marano@igb.cnr.it (D.M.); salvatore.fioriniello@igb.cnr.it (S.F.); maurizio.desposito@igb.cnr.it (M.D.)

**Keywords:** Rett syndrome, *MeCP2*, DNA methylation, epigenomics, non-coding RNAs, transcriptomics, histone modifications, chromatin

## Abstract

Rett syndrome (RTT) is an extremely invalidating, cureless, developmental disorder, and it is considered one of the leading causes of intellectual disability in female individuals. The vast majority of RTT cases are caused by de novo mutations in the X-linked Methyl-CpG binding protein 2 (*MECP2*) gene, which encodes a multifunctional reader of methylated DNA. MeCP2 is a master epigenetic modulator of gene expression, with a role in the organization of global chromatin architecture. Based on its interaction with multiple molecular partners and the diverse epigenetic scenario, MeCP2 triggers several downstream mechanisms, also influencing the epigenetic context, and thus leading to transcriptional activation or repression. In this frame, it is conceivable that defects in such a multifaceted factor as MeCP2 lead to large-scale alterations of the epigenome, ranging from an unbalanced deposition of epigenetic modifications to a transcriptional alteration of both protein-coding and non-coding genes, with critical consequences on multiple downstream biological processes. In this review, we provide an overview of the current knowledge concerning the transcriptomic and epigenomic alterations found in RTT patients and animal models.

## 1. Introduction

Rett syndrome [1] (RTT, Online MIM 312750) is a devastating X-linked postnatal neurodevelopmental disease that affects predominantly female individuals, with a frequency ranging from 1/10,000 to 1/15,000 live female births [2,3]. RTT is classified as an autism spectrum disorder (ASD) [4,5] and is considered one of the leading causes of intellectual disability in females [6]. Clinical symptoms of RTT, which appear generally after a period of apparent normal development during the first two years of life, include loss of acquired skills (e.g., communication and purposeful hand use) and speech, development of stereotypic hand movements, motor and social disabilities, autistic-like features, mental retardation, seizures, and respiratory dysfunctions [3,7,8].

More than 95% of patients with RTT carry loss of function mutations in the X-linked *Methyl-CpG-binding Protein 2* (*MECP2*) gene [9,10], encoding a key epigenetic modulator of transcription crucial for maturation of neurons and for nervous system functioning [11].

RTT clinical manifestation is quite variable, with dependence on various factors, such as the type of mutation in the *MECP2* gene, its location in the functional domains of the protein, and the degree of skewing of X chromosome inactivation [12,13]. Rare, atypical variants of RTT are caused by mutations in *cyclin-dependent kinase-like 5* (*CDKL5*) and *forkhead box G1* (*FOXG1*) genes. Patients carrying these mutations, which are often affected by a congenital form of the disease, can show early onset of seizures and sometimes preserved skills and speech [14]. This review is focused on epigenomic and transcriptomic alterations in RTT caused by mutations in the *MECP2* gene.

Male patients with RTT have rarely been described. Male individuals with a normal karyotype and carrying *MECP2* mutations found in females with a classical RTT phenotype show a severe clinical picture characterized by neonatal encephalopathy, and they usually die within the first year of life. Alternatively, the same mutations are responsible for classic RTT phenotype in males with somatic mosaicism or Klinefelter syndrome (47, XXY) [13].

Since its identification [15] and even more so after its association with RTT [9], MeCP2 has intrigued the scientific community. MeCP2 is ubiquitously expressed, but its levels are higher in the brain. In this tissue, MeCP2 is predominantly expressed in neurons as compared with glia [16], and its levels in neuronal cells reach nearly those of histone octamer [17]. Constitutive or brain-specific ablation of *Mecp2* in mouse recapitulate many aspects of RTT, and this emphasizes the crucial role of this factor in brain function [18,19]. Importantly, neurological defects due to absence of MeCP2 in mice can be rescued by restoration of the gene, establishing the principle of reversibility in RTT [20].

MeCP2 is an intrinsically disordered protein (IDP) in which more than 60% of the polypeptide chain lacks well-defined secondary and tertiary structures [21,22,23]. The presence of these disordered regions facilitates structural reorganizations and the consequent exposure of different motifs that allow the interaction of MeCP2 with different molecular partners. Despite the intrinsically disordered nature of MeCP2, six structurally distinct domains have been identified: the N-terminal domain (NTD), the methyl-binding domain (MBD), the intervening domain (ID), the transcriptional repression domain (TRD), the C-terminal domain α (CTDα), and the C-terminal domain β (CTDβ) [24,25].

Although RTT mutations have been found throughout the entire *MECP2* gene [13], most of them are located within the MBD or in the nuclear receptor co-repressor/silencing mediator for retinoid or thyroid-hormone receptors (NCoR/SMRT) co-repressor interaction domain (NID), which is in turn included in the TRD [26,27,28]. These mutations affect the ability of the protein to bind methylated DNA and to repress MeCP2 target genes, respectively. Noteworthy, recent findings highlighted that the expression of a truncated form of MeCP2, including just the MBD and the NID, rescues neurological phenotypes in *Mecp2*-deficient mice [29], thus suggesting that the primary function of MeCP2 is the recruitment of the NCoR/SMRT co-repressor complex to methylated DNA, and that alterations in this process are leading causes of RTT.

The *MECP2* gene encodes two differentially spliced isoforms, namely MeCP2-e1 and MeCP2-e2, which share the MBD, the TRD, and the C-terminal portion but exhibit a different N-terminus [30]. MeCP2-e1, which contains the exon 1, is the most abundant isoform in the brain in comparison with MeCP2-e2, which includes the exon 2 [31]. Many studies emphasized the predominant role of MeCP2-e1 isoform in RTT pathogenesis [32,33,34]. Recent findings underlined that MeCP2-e1 and MeCP2-e2 isoforms exhibit differential genomic occupancy and interact with different partners, which support the idea that, beyond the common roles in which both isoforms are involved, MeCP2-e1 and MeCP2-e2 have also different biological functions [35]. However, the functional overlapping of the two MeCP2 isoforms is still debated [36]. 

MeCP2 is involved in a plethora of biological processes, although its primary function is related to transcriptional repression. This action is accomplished through the binding of its MBD with methylated promoters of target genes [37,38] and the subsequent recruitment of histone deacetylases (HDACs) and co-repressors, through its TRD [28,38,39,40]. Nonetheless, MeCP2 can also act as an activator of gene expression [41,42,43] and as a post-transcriptional regulator, through the modulation of alternative splicing [44,45,46,47,48] and miRNA processing [49]. This multi-tasking ability might be due to the interaction of MeCP2 with co-factors that have alternative roles [30,50].

In addition to the well-known function of MeCP2 in the modulation of transcription of specific target genes, increasing findings support the idea that this multifaceted protein has also genome-wide functions [30,50]. MeCP2, indeed, shows a wide distribution in the mouse neuronal genome and its binding mirrors methyl-CG (mCG) and mCAC density [17,51,52,53], which indicates a role in the large-scale organization of the genome. In support of this hypothesis, MeCP2 loss in the brain has been associated with global changes in the epigenomic and transcriptomic landscape [17,42,53,54,55], which include higher levels of histone H1 and a global increased acetylation of histone H3 [17]. In addition, increased transcriptional noise has been observed in *Mecp2*-null neurons, thus underlining a role of MeCP2 in the repression of spurious transcription of repetitive elements [17]. Furthermore, MeCP2 is implicated in higher-order organization of pericentric heterochromatin, a considerable portion of the genome thought to form silent compartments in the nucleus [30,56,57,58,59,60]. Conversely, MeCP2 seems to be dispensable for the formation of self-interacting genomic regions, such as topologically associating domains (TADs) [61].

Altogether, the evidence collected so far pinpoints MeCP2 as a multi-tasking factor that, starting from the reading of DNA methylation in the genome, has a global impact on the coding and non-coding (nc) transcriptional landscape and on the expression of spurious elements, through the modulation of the epigenome, and on the organization of repressive nuclear compartments involved in gene silencing. In this scenario, MeCP2 dysfunctions causing RTT are responsible for large-scale derangements in neurons with a harmful effect on many biological processes, which will be detailed in the following sections. 

## 2. MeCP2: A Reader or a Regulator of DNA Methylome?

The functional role of MeCP2 is closely related to DNA methylation. The affinity of MeCP2 for methylated DNA has been known for many years [15]. However, nowadays, it is well established that MeCP2 binds both methylated and non-methylated DNA [62] with a strong enrichment in mCG-containing regions [17] and across mCH-rich sequences (where H = A, C, T) [51,53,61,63]. Moreover, according to its dual role as both a transcriptional activator and repressor [42], MeCP2 binds also 5-hydroxymethylcytosine (hmC) [51,64], one of the oxidized forms of mC [65] generally associated with active chromatin [66].

Increasing findings strengthen the importance of the diverse forms of DNA epigenetic modifications for the correct functioning of MeCP2 in the central nervous system. Several RTT mutations, indeed, are known to impair the binding of MeCP2 to methylated and/or hydroxymethylated DNA [64,67]. Moreover, conditional knock-out mice lacking the enzyme responsible for the establishment of mCH, the de novo DNA methyltransferase 3A (DNMT3A), in GABAergic inhibitory neurons, develop severe neurological phenotypes that include some symptoms observed in mice lacking *Mecp2* in the same neuronal sub-types [68]. Notably, transgenic mice that express an engineered MeCP2 protein able to bind mCG, but not mCH, display an RTT-like phenotype [69]. Altogether, this evidence remarks the relevance of both CG and non-CG DNA methylation in RTT pathogenesis.

In 2013, Zhao and coworkers proposed a novel role of MeCP2 in the regulation of alternative splicing in an intragenic DNA methylation-dependent manner [44]. According to this intriguing model, they demonstrated that the included alternatively spliced exons (ASEs) are hypermethylated and enriched in MeCP2, as compared with excluded ASEs, thus suggesting that MeCP2, through the binding of methylated DNA, promotes the exon inclusion. Moreover, this process seems to correlate with the kinetics of transcriptional elongation, mediated by RNA polymerase II, and with HDAC function [44]. These results underline the contribution of MeCP2 in the regulation of alternative splicing and add another piece to the intricated crosstalk between MeCP2, DNA methylation, and transcriptional regulation. It should be emphasized that the role of MeCP2 in the control of alternative splicing has been extensively supported by its physical interaction with different splicing factors, its association with exon/intron boundaries, which are significantly enriched in hmC and mCG, and by the aberrant alternative splicing observed in MeCP2-altered conditions [45,46,47,48]. However, the significance of MeCP2 and DNA methylation in the regulation of alternative splicing has recently been questioned [70].

In addition to the well-known ability of MeCP2 to act as a reader of DNA methylation, it is reasonable that this protein can, indirectly, participate in the establishment and/or maintenance of this epigenetic modification. Since 2003, it has been known that MeCP2 interacts with the maintenance DNA methyltransferase DNMT1 through the TRD, and that this interaction prevents its association with HDAC1 [71]. The MeCP2/DNMT1 interaction allows one to suppose involvement of MeCP2 in the DNMT1-mediated maintenance of DNA methylation. In addition, a recent work highlighted that MeCP2 binds DNMT3A, with a possible role in its physical recruitment and in the modulation of its catalytic activity. The binding of MeCP2 inhibits, indeed, the enzymatic activity of DNMT3A in vitro by stabilizing its closed, auto-inhibitory conformation. Moreover, overexpression of MeCP2 in the HCT116 human cell line led to a global reduction of DNA methylation levels [72]. Against this background, one would expect that, theoretically, upon MeCP2 dysfunction, as in the case of RTT, the regulatory effect that MeCP2 exerts on DNMT3A may be missing, leading to aberrant DNA methylation across the genome. However, the impact of MeCP2 malfunction on global DNA methylation and its large-scale effect in RTT has been poorly investigated and is still elusive, also considering that MeCP2 is pinpointed as an epigenetic reader, rather than a writer of DNA methylation. 

In a work published in 2013, it was postulated that disruption of MeCP2 function in RTT could give rise to compensatory changes in DNA methylation [73]. Genomic mCG profiles, obtained from lymphoblastoid cells of RTT patients and healthy controls, showed some differentially methylated regions, but these results were not confirmed when the experiments were performed on a larger cohort [73]. In the same year, an array-based methylome assay was performed in skin fibroblasts derived from RTT monozygotic twins displaying different disease manifestation and severity, even sharing the same mutation in *MECP2* and the same X-chromosome inactivation patterns. The authors found differential DNA methylation in 252 loci, 100 of which were hypermethylated and 152 of which were hypomethylated in the patient with the milder phenotype [74]. This evidence suggests that phenotypic differences observed in RTT patients sharing the same genotype could be mirrored by differences in the relative epigenomic landscape. However, considering that both twins carried the same *MECP2* mutation, additional factors are likely responsible for the observed changes in DNA methylation. Moreover, this study, performed only in two RTT patients, was mostly focused on gene promoters, and the coverage was ~2% of the human genome, thus leaving open the possibility of differential DNA methylation in other loci [74].

In a more recent study, mCG profiles have been analyzed in the Broadmann Area 9 of the cerebral cortex from six RTT patients through whole-genome bisulfite sequencing (WGBS), which revealed 2931 loci hypermethylated and 1975 hypomethylated in the RTT cohort. Differentially methylated regions (DMRs) were enriched in loci displaying an active chromatin state and were distributed in many functional elements including enhancers, promoters, as well as in exons and 3’ UTRs. Gene Ontology (GO) analysis revealed that DMRs were enriched in 483 GO terms, which include nervous system development, neurogenesis, synapses, cell morphogenesis, and processes associated with actin and cytoskeleton, and, when DMRs were assigned to the closest loci, they matched with several genes involved in ASDs. It is interesting to note that some of the DMRs found in RTT patients were associated with genes deregulated in the RTT brain [75]. 

**Figure 1:**
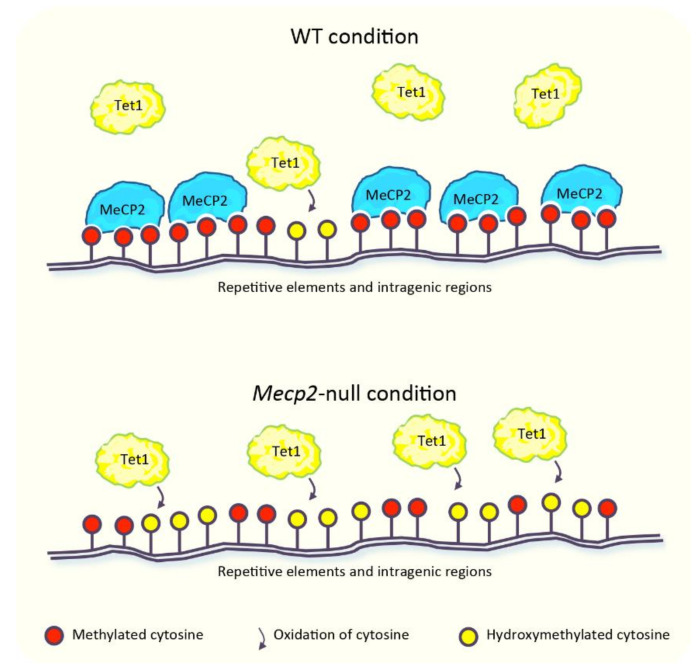
MeCP2 protects DNA methylation from oxidation. In physiological conditions, MeCP2 competes with Tet1 for the binding of methylated cytosines across repetitive elements and intragenic regions (**top**). The absence of MeCP2 opens the access to Tet1, which converts 5-methylcytosines to 5-hydroxymethylcytosines, leading to an enhanced hydroxymethylation (**bottom**) [76,77].

MeCP2 has been proposed to “survey” the genome by protecting mC from oxidation to hmC. According to this model, the binding of MeCP2 to methylated DNA could restrict the access of Tet1 [76,77], one of the enzymes responsible for the oxidation of mC [65] (Figure 1). Large-scale studies performed in murine cerebellum revealed that global hmC levels were negatively correlated with MeCP2 dosage [77]. The levels of hmC, indeed, were ~20% higher in *Mecp2*-null and ~25% lower in MeCP2-overexpressing mice, compared to the wild type (WT) animals. In agreement with the global increase of hmC in the *Mecp2*-deficient condition, this epigenetic modification displayed a higher enrichment across the intragenic regions of several loci and in repetitive elements, including long tandem repeat (LTR), short interspersed nuclear element (SINE), and long interspersed nuclear element (LINE) (Figure 1). This latter evidence allowed to hypothesize a contribution of hmC in the aberrant expression of these repeats observed in the absence of MeCP2 (detailed in Section 5) [77]. In support of this concept, enhanced accumulation of hmC at pericentric heterochromatin of *Mecp2*-null neurons parallels the increased transcription of major satellite DNA [76], which belongs to the class of murine repetitive DNA sequences [57]. The supposed role of MeCP2 in the protection of mC from uncontrolled oxidation [76,77] would explain, at least in part, the differences in DNA methylation observed in RTT patients [75]. However, the relation between MeCP2 and hmC is still debated. Some genomic regions dynamically modified, which gain or lose hmC during the development of the cerebellum, indeed display a lower accumulation of hmC in the *Mecp2*-null condition, in partial contrast with its overall increased levels [77]. Furthermore, in granule cells obtained from the cerebellum of *Mecp2*-null mice, a slightly lower accumulation of hmC in the gene body of some expressed and non-expressed genes has been observed, although its global genomic distribution is unchanged in comparison with the WT condition. In light of this, the authors proposed that MeCP2 does not contribute to the establishment of hmC [64].

To the best of our knowledge, the genomic distribution of 5-formylcytosine (fC) and 5-carboxylcytosine (caC), deriving from the further oxidation of mC/hmC [78] in RTT remain largely unexplored. Aberrant levels of fC and caC have been described in some human diseases [79,80]. Therefore, it would be interesting to investigate them in RTT patients and/or in RTT mouse models to gain further insights into the relationship between MeCP2 and the regulation of DNA methylation and its derivatives.

Overall, these findings raise the possibility that rather than being just a reader of diverse forms of DNA methylation, MeCP2 could itself participate indirectly to the refinement of these DNA epigenetic modifications, thus adding an additional layer of complexity to the MeCP2-mediated regulation of gene expression. However, the available data are still controversial and further studies are required to shed light on this aspect.

## 3. MeCP2: “The Guardian” of Neuronal Epigenome

MeCP2 is one of the most abundant nuclear proteins in neurons, with a number of molecules approaching that of histone octamers [17]. Due to its high expression levels and its extensive genomic distribution, MeCP2 has been proposed to modulate the configuration of the neuronal epigenome through the recruitment of several molecular partners [17,30,56,57,59]. With this evidence in mind, it is not surprising that MeCP2 dysfunction leads to extensive epigenomic alterations in neurons, which include the uncontrolled spreading of histone modifications, the altered recruitment of enzymatic factors, and the aberrant binding of proteins involved in the regulation of gene expression (described hereafter). 

In *Mecp2*-null conditions, the expression level of histone H1 is nearly doubled, and this phenomenon is mainly restricted to neurons [17]. Moreover, MeCP2 competes in vitro with histone H1 for the binding of chromatin containing methylated DNA [27]. These findings led researchers to hypothesize, for many years, that MeCP2 could coat the neuronal genome in vivo, thus replacing histone H1, which is in line with the lower histone H1 levels observed in neurons, in comparison with other cell types in which MeCP2 levels are lower [17,81]. However, a more recent work showed that the in vivo genomic binding of histone H1.0, a subtype of histone H1, does not change significantly in the absence of MeCP2, and that MeCP2 genomic distribution was unaltered upon overexpression of histone H1.0. Furthermore, both H1.0 and MeCP2 occupied some common genomic regions [82]. These results suggested that MeCP2 and histone H1.0 are largely independent for their binding to chromatin, in contrast to what was previously postulated. Thus, the relationship between MeCP2 and histone H1 remains still controversial so far, and whether the lower abundance of histone H1 in neurons can be ascribed to the higher levels of MeCP2 remains still an open question.

**Figure 2:**
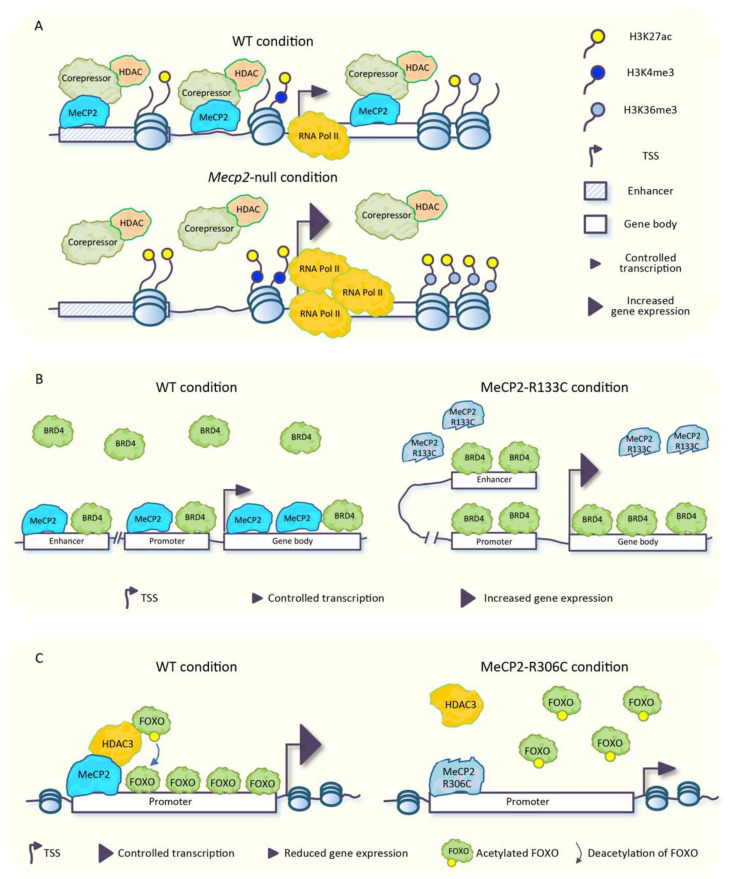
MeCP2 dysfunctions lead to uncontrolled distribution of epigenetic factors and histone modifications. (**A**) In WT neurons, MeCP2 controls gene expression by recruiting complexes containing histone deacetylase activity, which survey the acetylation status of H3K27 across enhancers, TSS, and gene bodies (top). The absence of MeCP2 leads to the enhanced acetylation of H3K27 across regulatory regions and gene body and causes a higher accumulation of RNA pol II at TSS. These changes are accompanied by a higher enrichment of H3K4me3 at TSS and of H3K36me3 at gene bodies, which correlate with gene up-regulation (bottom) (adapted from [61,83]). (**B**) In WT interneurons, in basal physiological conditions, the binding of BRD4 across enhancers, promoters, and gene bodies is low (left); in MeCP2-R133C interneurons, the binding of BRD4 across gene bodies and regulatory regions increases. These changes parallel a higher frequency of enhancer/promoter interactions with the resulting gene up-regulation (adapted from [84,85]). (**C**) In physiological conditions, MeCP2 recruits HDAC3, which deacetylates FOXO transcription factors [86]. Once deacetylated, FOXO proteins bind DNA and activate transcription (left) [87,88]. In MeCP2-R306C neurons, the increased acetylation of FOXO proteins occurs, which leads to the reduced enrichment of these transcription factors to promoters. These events lead to gene down-regulation (right) [86].

It is well established that MeCP2 mediates transcriptional repression through the recruitment of several repressive complexes containing HDACs and repressors/corepressors such as SIN3 transcription regulator homolog A (Sin3A), repressor element-1 silencing transcription factor-corepressor 1 (CoREST), and NCoR/SMRT, with the consequent deacetylation of histone tails [30] (Figure 2A). In line with this MeCP2 function, in neurons lacking MeCP2, a strong enrichment of total acetylated histone H3 across several genomic regions, and an increased transcriptional noise from repetitive elements have been found (see also Section 5). This evidence led some to presume that MeCP2 could protect the neuronal epigenome by restricting the uncontrolled spreading of histone acetylation, which might have detrimental effects for neuronal function [17]. This idea has been further corroborated in other studies. In the frontal cortex of mice carrying the MeCP2-R306C mutation, which disrupts the interaction of MeCP2 with NCoR co-repressor complex but not with DNA [28], a small increase of acetylation of histones H3-Lys27 (H3K27ac), H3-Lys9 (H3K9ac), and H4-Lys12 (H4K12ac) was found at the transcriptional start site (TSS) and at the gene body of genes that were up-regulated [61]. Moreover, in *Mecp2*-null mouse cortex, the stronger abundance of H3K27ac, observed at the TSS of up-regulated genes, parallels an increased enrichment of trimethylated H3-Lys4 (H3K4me3) in the same genomic regions and an increased methylation of H3-Lys36 (H3K36me3), another histone mark associated with active transcription, at the gene body of the same genes (Figure 2A). An opposite trend was observed in mice overexpressing MeCP2 [83]. Although changes in histone mark distribution are subtle, they are consistent with the magnitude of mRNA increases for these genes. Furthermore, in the *Mecp2*-null cortex, a higher accumulation of H3K27ac was also found across enhancers located near genes repressed by MeCP2, which is suggestive that in physiological conditions, MeCP2 promotes enhancer deacetylation to block their activating effects on target promoters (Figure 2A) [83]. Of note, TSS and enhancers of genes activated by MeCP2 were hypoacetylated in the *Mecp2*-null cortex [61,83]. 

The aberrant spreading of histone acetylation that takes place in the MeCP2-defective condition might have a harmful impact on the binding of the numerous readers of this type of epigenetic modifications, leading to an uncontrolled spreading of these factors across the genome. In agreement with this hypothesis, the abnormal binding of Bromodomain protein 4 (BRD4), a reader of histone acetylation that promotes gene transcription by enhancing chromatin accessibility [84], has been observed across the genome of human embryonic stem cell (hESC)-derived interneurons (INs) carrying the MeCP2-R133C RTT mutation. BRD4 was, indeed, aberrantly accumulated at enhancers, promoters, and transcribed regions of a large number of genes (Figure 2B). Moreover, in these mutated cells, the genomic binding of BRD4 was not responsive to KCl-induced depolarization as, instead, occurs in physiological conditions, which results in a permanent hyperactive state accompanied by up-regulation of several genes. Furthermore, in the same cellular model, several abnormally open chromatin regions (OCRs) enriched in BRD4 have been identified at distal enhancers of differentially expressed genes. In addition, mutant cells displayed an increased interaction between enhancers and promoters (Figure 2B) [85], which is in line with the aberrant activation of these regulatory elements observed in *Mecp2*-null mouse brain [61,83].

Interestingly, treatment of MeCP2-R133C INs with JQ1, an epidrug that targets primarily BRD4, reduced the genome-wide occupancy of BRD4 in basal conditions, restored the spreading of BRD4 genomic binding upon KCl stimulation, and normalized the expression of up-regulated genes, including some immediate-early genes induced by depolarization [85]. Moreover, JQ1 treatment rescued the aberrant open chromatin state. Furthermore, JQ1 treatment increased the lifespan and ameliorated the pathological phenotype of *Mecp2*-null mice. Altogether, these findings suggested that the aberrant genome-wide binding of BRD4 is responsible, at least in part, for the transcriptional alterations that takes place in cases of MeCP2 malfunctions, with a plausible impact on the RTT phenotype in vivo [85]. However, whether the aberrant genome-wide binding of BRD4 depends on the abnormal enrichment of histone acetylation is still obscure, since the global distribution of this type of epigenetic modifications in MeCP2-R133C INs has not been investigated. Nevertheless, the MeCP2-R133C mutation is known to impair the binding of MeCP2 to DNA (Figure 2B) [67,85]; thus, it is tempting to speculate that the missed chromatin binding of MeCP2 could lead to a reduced recruitment of HDACs and the subsequent accumulation of histone acetylation which, in turn, acts as a binding platform for BRD4. 

In addition to its role as a reader of acetylated histones, BRD4 acts also as a histone acetyltransferase (HAT) of histones H3 and H4 [84]. This evidence allows one to suppose that the uncontrolled spreading of BRD4 across the genome might contribute also to the unbalanced distribution of histone acetylation observed in the MeCP2-defective context, with a positive feedback-loop mechanism. However, further studies are needed to address this point.

The crosstalk between MeCP2, histone acetylation, and gene expression becomes more intricate when considering that in neurons, MeCP2 promotes the expression of a number of genes through the association with HDAC3 [86], a HDAC included in the NCoR corepressor complex, known to interact with MeCP2 [28]. Mice lacking *Hdac3* in forebrain excitatory neurons (*Hdac3* conditional knock-out; *Hdac3*-cKO) showed, in the hippocampal CA1 region, deregulation of several genes, a sub-set of which were coherently down-regulated in *Mecp2*-null hippocampus. Of note, the binding of HDAC3 to the TSS of several genes deregulated in the *Mecp2*-null hippocampus was reduced in the absence of MeCP2, thus suggesting a function of MeCP2 in the recruitment of HDAC3 to these regions. Tsai and coworkers [86] investigated the complex mechanisms underlying the transcriptional regulation mediated by MeCP2 and HDAC3. They found that genes regulated by both MeCP2 and HDAC3 contain the binding motif for the FOXO transcription factors. The ability of FOXO proteins to promote gene expression [87,88] is, in turn, inhibited by their acetylation, a process that is counteracted by HDAC3 [89]. They found that FOXO acetylation increased in the absence of HDAC3 or MeCP2 in murine hippocampal neurons and in iPSCs-derived neural progenitor cells (NPCs) from RTT patients carrying the MeCP2-R306C mutation (Figure 2C) [86]. The authors hypothesized that MeCP2-loss is responsible for decreased recruitment of HDAC3, with the consequent increased FOXO acetylation [86], and this results in reduced gene activation. Of note, the ablation of *Hdac3* in forebrain excitatory neurons causes a number of neurological phenotypes observed in forebrain-specific *Mecp2* cKO mice [90], which allows one to hypothesize a contribution of HDAC3 dysfunction to RTT pathogenesis in vivo [86].

Taken together, the aforementioned evidence suggested that MeCP2 exploits a double mechanism to regulate transcription via HDACs, with opposite consequences. On the one hand, MeCP2 targets HDACs to certain genomic regions to promote deacetylation of histone tails, with the following transcriptional repression (Figure 2A); on the other hand, MeCP2 recruits HDAC3 to induce deacetylation of FOXO proteins, which, in turn, promote gene expression (Figure 2C). This latter mechanism, together with the known interaction of MeCP2 with CREB1 [42] would explain, at least in part, the down-regulation of a plethora of genes observed in MeCP2-altered conditions, despite the well-known repressive function of MeCP2 (detailed in the Section 5).

It is worth noting that in physiological conditions, a large number of non-histone proteins are dynamically acetylated and deacetylated by the action of different HATs and HDACs, respectively. Among them are chromatin remodeling complexes, splicing factors and enzymes that refine the chromatin epigenetic landscape, such as histone demethylases and HATs. Upon acetylation, some transcription factors (including FOXO) display a reduced ability to bind DNA and then, to promote gene expression, whereas others bind DNA with a higher affinity. Furthermore, the acetylation of a certain transcription factor can have different functional outputs, depending on the modified amino acid residue [91,92]. In this frame, it is reasonable to speculate that the missed recruitment of HDACs across the genome, in the MeCP2-defective condition, perturbs the balance between acetylation and deacetylation of many factors, which leads to their abnormal functioning, as observed for FOXO proteins, with a possible impact on RTT pathogenesis. 

## 4. MeCP2 Is a Genome-Wide Modulator of Chromatin Architecture in Neurons

**Figure 3:**
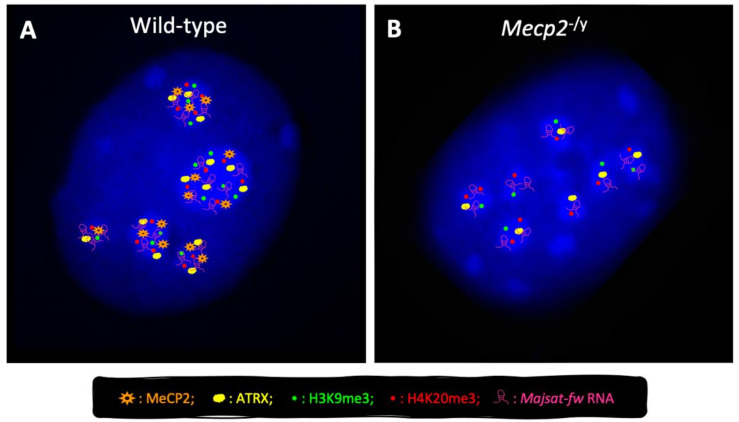
Schematic representation of pericentric heterochromatin organization in nuclei of wild-type and *Mecp2^−^*^/y^ neurons derived from differentiation of murine embryonic stem cells. Representative images of interphase wild-type (WT) (**A**) and *Mecp2*^−/y^ (**B**) nuclei were depicted. Chromocenters are highlighted by intense 4′,6-diamidino-2-phenylindole (DAPI) staining (blue). In terminally differentiated *Mecp2*^−/y^ cells (**B**), the number of chromocenters is higher and their size is lower in comparison with WT cells (**A**), due to the role of MeCP2 in the pericentric heterochromatin condensation (chromocenter clustering) during neural differentiation [60]. Moreover, MeCP2 contributes to the targeting of ATRX [59] and *MajSat-fw* transcript to chromocenters and preserves the correct deposition of H3K9me3 and H4K20me3 histone marks to pericentric heterochromatin [56].

The role of MeCP2 in the modulation of genome architecture and its ability to compact heterochromatin in neurons have been largely demonstrated, as well as the involvement of other proteins and ncRNAs that cooperate with MeCP2 in this process [30,56,57,58,59,60]. It has long been known that in mouse cells, MeCP2 accumulates at pericentric heterochromatin (PCH) [15], a large fraction of constitutive heterochromatin containing hypermethylated major satellite repeats, which is organized in higher-order structures called chromocenters [57] (Figure 3A). PCH is an epigenetically well-defined entity with key roles in chromosome segregation and genome stability, which is thought to form repressive nuclear compartments because of the local concentration of silencing factors [57,93,94,95].

Increasing reports highlight a key role of MeCP2 in PCH condensation (chromocenter clustering) during both myogenic and neural differentiation [30,56,57,96,97] (Figure 3). We recently highlighted a contribution of Alpha-thalassemia/mental retardation syndrome X-linked (ATRX) protein [59] in MeCP2-mediated chromocenter clustering in neurons. This is of particular interest, considering that ATRX is a chromatin remodeling factor mutated in ATR-X syndrome [98,99], another disease associated with severe intellectual disability. Furthermore, we highlighted that MeCP2 and ATRX promote their reciprocal expression and their targeting to chromocenters in neurons (Figure 3), and that both factors modulate the expression of members of the heterochromatin protein 1 (HP1) family [59], proteins that are highly enriched in PCH.

Of note, several RTT-causing mutations located within the MBD of MeCP2 impair its accumulation at PCH and affect chromocenter clustering [56,97,100,101], which allows one to suppose an impact of these defects in RTT pathogenesis.

We have recently demonstrated, for the first time, the cooperation between MeCP2 and the major satellite forward (*MajSat-fw*) transcript, a ncRNA encoded by major satellite repeats, for the higher-order PCH organization in neurons. These two factors, indeed, physically interact with each other and are mutually dependent for their targeting to PCH (Figure 3). These findings reinforce the idea that MeCP2 and *MajSat-fw* RNA act as structural organizing factors of the silent compartments in neuronal PCH, where they participate in the maintenance of the heterochromatic status [56]. Moreover, we underlined the prominent role of MeCP2-e1 in both chromocenter clustering and *MajSat-fw* RNA recruitment to chromocenters, the relevance of MBD for PCH condensation, and the requirement of both MBD and TRD for *MajSat-fw* targeting to chromocenters [56]. Lastly, we observed that the MeCP2-T158M RTT mutation, located within the MBD, affects higher-order PCH organization in neurons [56], which underlines the importance of the MBD for this function.

The contribution of MeCP2 to the global organization of PCH architecture is further supported by its ability to preserve the correct deposition of two repressive histone marks, trimethylated H3-Lys9 and H4-Lys20 (e.g., H3K9me3, H4K20me3), to PCH in neurons (Figure 3) [56], and by its role in the recruitment of HP1 to chromocenters during myogenic differentiation [102]. It is conceivable that reduced targeting of *MajSat-fw* RNA to chromocenters caused by MeCP2 dysfunction leads to defective recruitment of suppressor of variegation 3–9 homolog (Suv39h) and suppressor of variegation 4–20 homolog (Suv420h) histone methyltransferases (HMTs), and then to reduced H3K9me3 and H4K20me3 deposition at PCH, according to the known function of satellite transcripts to recruit Suv39h to PCH [103,104].

So far, the role of MeCP2 in the organization of PCH in human cells is still unclear. However, it has been reported that MeCP2 over-expression induces heterochromatin clustering also in human fibroblasts [97]. 

Taken together, these studies provide compelling evidence of a key role of MeCP2 in global chromatin architecture and in the organization of silent nuclear compartments in neurons. In this regard, one can speculate that the altered structure of repressive nuclear compartments, caused by MeCP2 dysfunctions, affects the appropriate nuclear localization of specific genes, with their resulting incorrect expression, and that this might have an impact in RTT pathogenesis. However, additional studies are needed to confirm this fascinating hypothesis.

An interesting perspective that is recently emerging is the role of MeCP2 in liquid–liquid phase separation (LLPS) [105,106]. LLPS has been proposed as a novel mechanism involved in the formation of membrane-less cellular compartments with well-defined chemical properties, in which diverse biochemical reactions can occur [107]. Intracellular phase transitions originate from weak and transient interactions between multiple proteins, often characterized by an intrinsically disordered structure [108,109]. Nuclear phase transition is of particular relevance, considering that nuclear compartments can control chromatin architecture and, then, gene expression. The formation of heterochromatin domains seems to be dependent, at least in part, on LLPS [110,111,112,113,114]. In this context, two recent studies highlighted that MeCP2, in complex with DNA, promotes LLPS [105,106], according to its intrinsically disordered nature. Li and coworkers [106] demonstrated that in vitro, at physiological salt conditions, the combination of MeCP2 and nucleosomal arrays (or DNA alone) leads to the formation of numerous *puncta*, thought to be driven by phase separation, that are reminiscent of MeCP2-associated heterochromatin domains observed in vivo [106]. The authors suggested that MeCP2 induces LLPS through its homodimerization and interaction with DNA and other molecular partners, thanks to its intrinsically disordered nature [106]. Of note, MeCP2-mediated LLPS is compromised in the presence of different RTT mutations [105,106], thus raising the possibility that defects in this process may contribute to RTT pathogenesis. Nevertheless, further investigations are required to better clarify this point.

## 5. The Transcriptomic Landscape Is Globally Altered in RTT

### 5.1. MeCP2 Is a Fine-Tuning Modulator of Transcription

**Figure 4:**
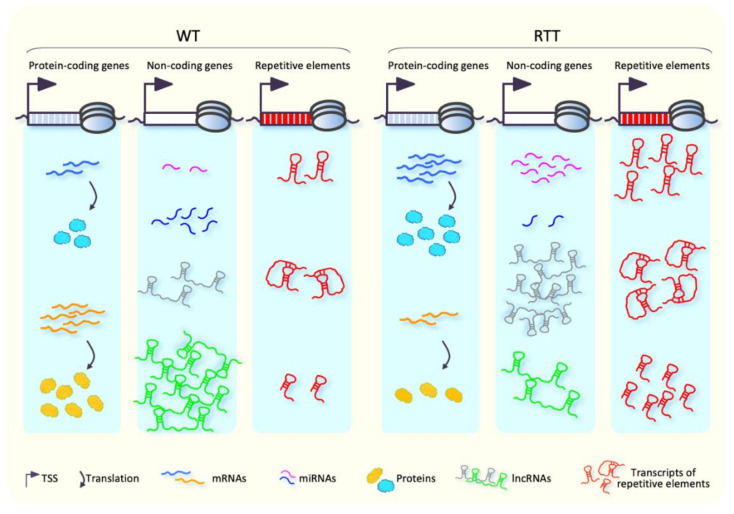
MeCP2 dysfunctions lead to a global alteration of the transcriptional program. (**Left panel**): In physiological conditions, both protein-coding and non-coding genes give rise to a variety of transcripts, some of which are expressed at high levels, whereas others are less abundant. Furthermore, the transcription rate of repetitive elements (including LINEs, intracisternal A particles and satellite DNA) is generally low. (**Right panel**): MeCP2 dysfunctions lead to the unbalanced production of both protein-coding and non-coding RNAs in both directions and to a general increased expression of repetitive elements.

For many decades, MeCP2 has been known to be a global epigenetic regulator of gene expression. Alterations of transcriptomic landscape in both RTT patients and murine models have been, indeed, widely reported during recent years [17,42,52,55,61], although direct evidence that links the observed gene mis-regulation with RTT physiopathology is still elusive. Initial investigations of transcriptional profiles performed in whole brain of RTT patients and mouse models revealed only small changes in gene expression [115,116,117]. Afterwards, studies carried out in specific brain regions and cell types highlighted deregulation of several genes in MeCP2-altered conditions (Figure 4), including genes implicated in neuronal function [41,42,43,52,53,55,118], which suggested a brain region- and cell type-specific function of MeCP2 in the regulation of gene expression. 

MeCP2 is thought to act primarily as a transcriptional repressor in a DNA-methylation dependent manner [19,27,38]. However, loss of MeCP2 function in different brain regions is responsible for both up- and down-regulation of thousands of genes (Figure 4), which seems to indicate a dual role for MeCP2 as both activator and repressor of transcription [41,42,53]. Of note, the magnitude of gene expression changes observed in the MeCP2-altered conditions is moderate, which pinpoints MeCP2 as a fine-tuning modulator of gene expression. 

Additional findings support a role of MeCP2 as a transcriptional activator. In both human and murine MeCP2-deficient neurons, indeed, the global RNA amount and the transcriptional capability are reduced [42,53,119,120]. However, the direct involvement of MeCP2 in the positive regulation of transcription is still controversial. On one hand, the direct binding of MeCP2 to the promoter of down-regulated genes in hypothalamus, its co-immunoprecipitation with the transcription factor CAMP responsive element binding protein 1 (CREB1), and the co-occupancy of both MeCP2 and CREB1 at the promoter of a representative activated gene, allow one to hypothesize that MeCP2 directly promotes the expression of a sub-set of genes in specific brain regions [42]. On the other hand, more recent studies that correlated MeCP2 genome occupancy and transcriptomic profiles in WT, *Mecp2*-null, and MeCP2 over-expressing (OE) mice showed the highest enrichment of MeCP2 across genes found up-regulated in *Mecp2*-null and down-regulated in MeCP2 OE mice, as compared with genes with an opposite behavior [51,53,119,121]. This latter evidence allows one to speculate that down-regulation of genes observed in the *Mecp2*-deficient condition is an indirect effect of MeCP2 dysfunction and emphasizes that the main role of MeCP2 is to promote transcriptional repression. In this regard, a recently proposed model suggests that MeCP2 serves as a bridge between methylated DNA and the NCoR/SMRT co-repressor complex, which contains HDACs and Sin3A. MeCP2 mutations in the MBD or NID, which impair the formation of this bridge, are sufficient to cause RTT, and the expression of a radically truncated form of MeCP2, containing only MBD and NID, reverts neurological symptoms in *Mecp2*-null models [28,29,122], which underlines the importance of the MeCP2-mediated transcriptional repression for neuronal function. Of note, the interaction between MeCP2 and NCoR complex is regulated by the activity-dependent phosphorylation of MeCP2-T308 amino acid residue [123]. 

To date, the mechanism by which MeCP2 regulates gene expression and the relevance of DNA methylation in this process have not yet been fully clarified. In particular, how mCG and non-mCG contribute to the MeCP2-mediated gene regulation is still debated. In the neuronal genome, MeCP2 is strongly accumulated in regions containing mCG and mCH [51,53,61,63] through its MBD, and this phenomenon appears to be the primary epigenetic feature that drives gene repression [52,53,61,69,119]. In this regard, the binding of MeCP2 to mCA, and especially to mCAC, seems to play a prominent role over mCG in gene regulation. Mice expressing an engineered form of MeCP2 able to bind mCG but not mCH showed, indeed, altered expression of several genes, one-third of which were similarly deregulated in *Mecp2*-null mice. Of note, most of these genes were associated with neurological diseases, which suggests their contribution to RTT pathogenesis [69]. Noteworthy, considering that non-CG methylation increases during postnatal neuronal maturation, as well as MeCP2 levels [16,17,66,124], the MeCP2-mediated gene regulation through the binding of mCH might explain the delayed onset of RTT [52,68,119].

The long genes highly methylated at CA sites within the gene body have been reported to be up-regulated in both RTT patients and murine models [51,53,55,61,83,119]. In addition, MeCP2 seems to repress the expression of these highly methylated long genes in a cell-type specific manner; this mechanism is cell-autonomous and agrees with the cell-type specific DNA methylation pattern [55]. Nonetheless, the role of MeCP2 in repressing the expression of highly methylated long genes in brain has been questioned. Levels of nascent transcripts derived from long genes were, indeed, unchanged in cortical neurons of MeCP2-R106W and MeCP2-T158M RTT mouse models. In this respect, it was proposed that MeCP2 might control levels of mature long mRNAs through a not yet well-characterized post-transcriptional mechanism [125]. Alternatively, the preferential up-regulation of long genes observed in MeCP2 altered conditions might be explained by the occurrence of artifacts during library preparation for RNA sequencing (RNA-seq) or microarray experiments [126]. However, in a more recent study, the selective repression of methylated long genes mediated by MeCP2 has been confirmed on both nascent and mature transcripts on multiple biological replicates [61].

The molecular mechanism by which MeCP2 represses the expression of long genes has been further investigated. At first, it was proposed that the binding of MeCP2 in the gene body, enriched in mCH, hampers the RNA polymerase II-mediated transcript elongation by acting as “speed bumps” [119,121]. Afterward, it was observed that up-regulated genes display a stronger enrichment of RNA polymerase II at the TSS and increased transcriptional initiation rate. This evidence suggests that MeCP2, through its interaction with NCoR co-repressor complex, modulates the expression of highly methylated long genes by hindering the transcriptional initiation, rather than elongation [61]. In physiological conditions, these genes showed a high rate of TSS-gene body contacts, which were not significantly altered in the absence of MeCP2 [61]. These structures may help the positioning of MeCP2 and NCoR co-repressor complex on the TSS of certain genes to repress transcription initiation [61]. Moreover, a parallel mechanism has also been suggested by which the binding of MeCP2 to mC in intragenic enhancers of genes up-regulated in RTT reduces the ability of these elements to activate the transcription of the target gene [83].

Overall, the findings collected in the last decades suggest a relevant role of the DNA methylation signature in MeCP2-mediated gene regulation. However, further studies are needed to better decipher the underlying molecular mechanisms.

### 5.2. Transcriptional Deregulation Affects Many Biological Processes in RTT Brain

The study of pathways altered in RTT, and the understanding of the underlying molecular mechanisms is pivotal to identify new molecular targets for the design of focused therapies to cure or ameliorate clinical symptoms.

The deregulation of a large number of coding and non-coding genes in RTT patients and mouse models (Figure 4) results in the alteration of several biological pathways, most of which are related to intracellular signaling and metabolism [127]. However, the comparison of transcriptomic profiles of different RTT samples often provides confounding results due to the different nature and age-stage of the sample analyzed. For this reason, the identification of discrete target pathways responsible for RTT pathogenesis is, currently, a challenge [127,128].

Perturbation of different biological processes in RTT has been reported, including those associated with lipid metabolism, immune response, mitochondrial function, synaptic plasticity, and neuronal functioning and maintenance.

Deregulation of many genes that modulate lipid metabolism in RTT patients has been found in both central nervous system [129,130,131,132] and plasma [133,134,135]. The analysis of transcriptomic profiles in different brain regions of *Mecp2*-null mice highlighted a deregulation of several gene encoding factors with a function in cholesterol metabolism [54,127,129,136,137,138]. Cholesterol is one of the most abundant lipids in the brain, where it is crucial for synaptic plasticity, neurite outgrowth, synaptogenesis, and myelination [139]; thus, it is conceivable that alteration of its levels in the brain has dramatic consequences. In this regard, down-regulation of the *farnesyl diphosphate farnesyltransferase* (*Fdft1*) gene, which encodes the first enzyme involved in cholesterol biosynthesis, and of the *squalene monooxygenase* (*Sqle*) gene, encoding a key rate-limiting enzyme of cholesterol synthesis, have been reported in brain of *Mecp2*-null animals [136,137,138]. In partial contrast with these data, a mutagenesis suppressor screen performed in *Mecp2*-null mice highlighted that a loss-of-function mutation in *Sqle* ameliorates the RTT-like phenotype, suggesting that perturbations of cholesterol synthesis can improve RTT symptoms [129]. According to this hypothesis, a treatment with statins ameliorated RTT phenotypes in *Mecp2*-null mice in terms of motor performance and lifespan [129]. Although alterations of peripheral and brain cholesterol levels have been found in both RTT patients and animal models [129,134,137,140], the link between MeCP2 dysfunctions and altered cholesterol metabolism is still controversial, also considering that a more recent study failed to detect improvements of the RTT-like phenotype in *Mecp2*-null mice upon treatment with statins [137,141]. 

Recently, a function of MeCP2 in the regulation of genes encoding factors involved in glycosphingolipid (GSL) metabolism (glycogenes) in gastric cancer cells has been proposed [142]. Although this cellular context is far from RTT, GSLs are glycosylated lipids strongly enriched in brain and are crucial for neuronal functioning [143]. This evidence prompts one to speculate that similar gene mis-regulation in brain might have a pathogenetic impact in RTT. In support of this hypothesis, decreased levels of GD1a and GT1b gangliosides, the most abundant GSLs in brain [143], have been detected in total brain, cerebellum, and cerebrospinal fluid of RTT patients [130,131,132]. However, to date, these data are still preliminary and controversial [144]. In addition, mutations in glycogenes have been described in several neurological disorders, and in particular, loss of function mutations in the *ST3GAL5* gene, which encodes a key enzyme for ganglioside biosynthesis, have been reported in two patients with an RTT-like phenotype [145]. A recent work further supports the idea of a contribution of lipid metabolism dysfunctions in RTT physiopathology. Profiles of several lipids involved in sphingolipid metabolism have been, indeed, found to be altered in blood of RTT patients carrying *MECP2* mutations [146]. Altogether, this evidence suggests that perturbations of lipid metabolism might participate to RTT clinical manifestation. However, this aspect is still far from being fully elucidated. 

MeCP2 is crucial for neuronal maturation. Its key role in neuronal function and maintenance, as well as synapsis formation and functioning during the post-natal stage has been widely described [147]. Accordingly, neuronal defects, in terms of decreased number of synapsis and dendritic complexity, have been found in brain of both RTT patients and mouse models [148,149]. Aberrant expression of genes associated with synaptic functions, hippocampal dendrite development, and abnormal neuronal excitatory and inhibitory activity in brain of *Mecp2*-null mice have been widely reported [43,150,151]. Moreover, deregulation of genes encoding post-synaptic membrane proteins, such as ion channels, synaptic scaffolding proteins, and ionotropic glutamate receptors have been shown in both inhibitory and excitatory cortical neurons of male and female MeCP2-T158M and MeCP2-R106W mice [125]. This evidence agrees with the central role of MeCP2 in neuronal function and with the neurological nature of RTT.

For many years, it has been known that MeCP2 regulates the expression of brain-derived neurotrophic factor (*Bdnf*), encoding a protein important for neuronal development, synaptic transmission, and neuronal plasticity [152]. *Bdnf* is one of the genes the expression of which has been found coherently down-regulated in both RTT patients and mouse models [42,55,152,153,154]. However, the mechanisms by which MeCP2 regulates *Bdnf* expression have been extensively disputed, and two models of action with an opposite direction (MeCP2-mediated activation or repression of *Bdnf* expression) have been proposed. More recent findings allowed researchers to build an intriguing “dual operation” model that integrates the two previously proposed mechanisms. This new view supports the idea that MeCP2 can dynamically act as both an activator and a repressor of *Bdnf*, with dependence on different factors, such as post-translational modifications, interaction with different molecular partners, and the epigenetic signatures (reviewed in [152]).

Conditional knock-out of *Bdnf* in post-mitotic neurons recapitulates many phenotypic aspects of RTT, whereas increased BDNF levels in brain of RTT mice ameliorate their pathologic phenotype [153,155,156], which suggest a critical impact of BDNF dysfunction in RTT physiopathology. Noteworthy, the involvement of BDNF in the development of GABAergic neurons and synapses has long been known [157]. In light of this, the impairment of BDNF signaling in MeCP2-altered conditions can be responsible for the altered formation and maturation of synapses in GABAergic neurons, which results in an excitatory/inhibitory imbalance, observed in *Mecp2*-deficient mice [158].

Increasing literature supports the impact of perturbations on the NF-κB signaling pathway in RTT pathogenesis. This pathway regulates many neuronal functions, such as dendritic complexity and neurite growth. In both peripheral blood lymphomonocyte cells (PBMCs) from RTT patients and in *Mecp2*^–/y^ mouse brain, an up-regulation of NF-κB signaling has been observed [159,160], which results in the over-expression of genes regulated by NF-κB downstream in the pathway, such as *tumor necrosis factor* (*Tnf*) and *Calcium/Calmodulin Dependent Protein Kinase II Delta* (*Camk2d*) [159]. Furthermore, increased expression of *Interleukin-1 receptor-associated kinase 1* (*Irak1*), encoding a key protein kinase involved in activation of NF-κB signaling pathway, has been reported in different brain regions of *Mecp2*-null mice [151,159,161]. Aberrant expression of genes regulated by NF-κB has also been found in primary cultured astrocytes from *Mecp2*^308/y^ mice [162]. The impact of deregulation of the NF-κB signaling pathway in RTT pathogenesis is further supported by the evidence that *Irak1* over-expression causes impairment of dendritic-circuit complexity in cortical neurons, and that the genetically-induced correction of the aberrant NF-κB pathway in *Mecp2*-null animals ameliorates the dendritic morphogenic phenotype and lifespan [159]. This evidence identifies the NF-κB signaling pathway as a potential new therapeutic target for RTT.

A number of genes associated with the immune response have been found deregulated in mouse models of RTT [116], in line with an alteration in immune and inflammatory response observed in both RTT patients and mouse models [163]. Moreover, a MeCP2-mediated transcriptional regulation of genes involved in microglia response to inflammatory and stress stimuli [164,165] has been proposed, according to the pathological role of defects in microglia in RTT [166,167].

Another aspect that for many years has intrigued the scientific community is represented by mitochondrial dysfunctions in RTT. Mitochondrial alterations have been described in brain of both *Mecp2*-null models and RTT patients [148,168,169]. These defects can be reasonably ascribed to the altered expression of genes involved in mitochondrial function [170,171,172]. Transcriptomic analysis of PBMCs from RTT patients, indeed, highlighted deregulation of genes encoding factors related to the function and the organization of mitochondria [173]. Moreover, increased expression of the *ubiquinol-cytochrome c reductase core protein 1* (*Uqcrc1*) gene, which encodes a subunit of mitochondrial respiratory complex III, has been positively correlated with the severity of symptoms in *Mecp2*-null mice, and this up-regulation paralleled the increased mitochondrial respiratory activity [169]. Moreover, down-regulation of *cytochrome c oxidase subunit 1* (*MTCO1*) gene, encoding the main subunit of the catalytic core of cytochrome C oxidase (COIV), has been reported in both the frontal cortex of RTT patients [168] and in skeletal muscle of *Mecp2*-null mice [174], with a resulting reduction of COIV enzymatic activity. Additionally, the role of MeCP2 in the regulation of genes encoding mitochondrial transcription factors (i.e., *NR3C1*) and mitochondrial ribosomal proteins (i.e., *MRPS33*) has been hypothesized in induced pluripotent stem cells (iPSCs) derived from RTT patients [172]. Considering that mitochondria represent the major source of reactive oxygen species (ROS) and that their dysfunction has been associated with increased oxidative stress and ROS production [173,175], defects in mitochondrial activity in RTT might represent one of the leading causes of oxidative stress, which has been extensively reported in RTT patients and murine models [170,176,177]. 

### 5.3. Levels of Non-Coding RNAs Are Widely Altered in RTT

Alterations in microRNA (miRNA) expression levels have been observed in brains of RTT patients, *Mecp2*-null models, and in neurons obtained from RTT-patient-derived iPSCs [178,179,180,181,182,183] (Figure 4). MicroRNAs are a class of small ncRNAs with an important role in the regulation of gene expression at post-transcriptional levels, through the binding of 3′-UTR of protein-coding mRNAs and the subsequent repression of their translation, or promotion of their degradation [184]. In this light, transcriptional deregulation of miRNAs, observed in RTT patients and animal models, might contribute to neurological manifestations of this disease by influencing protein output from mRNAs in brain. This hypothesis is consistent with the well-known role of miRNAs, as well as other ncRNAs, for the proper central nervous system functioning [185,186,187,188,189,190,191]. 

The analysis of the expression profiles of mature miRNAs in the cerebellum of early-symptomatic *Mecp2*-null mice revealed up- or down-regulation of ~17% of analyzed mature miRNAs, many of which are transcribed from the *Dlk1*-*Gtl2* imprinting domain [183]. The binding of MeCP2 within this cluster suggests a direct role of MeCP2 in transcriptional repression of the corresponding primary miRNAs (pri-miRNAs) [183]. Remarkably, since miRNAs expressed from this cluster are important for neuronal maturation [190,192], their contribution in neurological disorders, such as RTT, has been suggested [183]. 

In a similar study, the deregulation of several miRNAs has been shown in total brain of *Mecp2*-null mice [182]. Among them, miR-29 and miR-146, two small transcripts important for neural and glial processes [193,194], were found up-regulated and down-regulated, respectively [182].

Nowadays, two different mechanisms by which MeCP2 regulates miRNAs levels have been characterized. On one hand, MeCP2 binds miRNA promoter by acting as a direct transcriptional modulator [195,196,197]; on the other hand, it acts post-transcriptionally by influencing pri-miRNA processing [178,198,199]. 

Among miRNAs deregulated in RTT, miR-137, miR-15a, miR-184, miR-7b, and miR-134 have been found up-regulated in RTT mouse and cellular models [178,181,195,196,197]. It is important to note that miR-137, miR-15a, miR-7b, and miR-134 are involved in dendritic maturation [195,196,200].

It has been proposed that in physiological conditions, MeCP2 represses the expression of the primary miR-137, miR-15a, miR-184, and miR-7b through the binding of methylated CGs in their promoter [181,195,196,197]. In 2014, an alternative mechanism by which MeCP2 modulates miRNA levels was characterized. Qiu and coworkers [49] demonstrated, indeed, a role of MeCP2 in the control of miRNA biogenesis in brain, by hampering nuclear miRNA processing, through the interaction with DiGeorge syndrome critical region 8 (DGCR8). According to this model, increased levels of mature miR-134 in hippocampus of a *Mecp2*-null model are associated with enhanced pri-miR-134 processing [178]. 

Nakashima and coworkers demonstrated that MeCP2 facilitates the processing of miR-199. Of note, decreased levels of this miRNA lead to up-regulation of mTOR signaling, a biological pathway defective in RTT [199].

Recently, Levy and coworkers [198] suggested a further model of MeCP2-mediated miRNA regulation. According to this model, the binding of MeCP2/DGCR8 complex to miRNA loci enriched in mCG slows the RNA Polymerase II-mediated transcriptional elongation. This promotes the interaction of Drosha and DGCR8 with nascent pri-miRNAs for their processing. 

Accordingly, decreased DNA methylation in miRNA loci, and the consequent loss of MeCP2 binding, reduces the production of mature miRNAs, due to an increased rate of RNA Polymerase II-mediated elongation and the subsequent inability of Drosha to bind pri-miRNA [198]. 

The evidence that MeCP2 regulates the production of several mature miRNAs introduces an additional layer in the scenario of MeCP2-mediated transcriptional regulation. Thus, it is conceivable that MeCP2 can modulate the expression of target genes also through an indirect mode, by controlling the production of miRNAs that, in turn, target mRNAs found deregulated in RTT. According to this model, *Bdnf* transcript is a target of miR-30a/d, miR-381, and miR-495, which are up-regulated in the cerebellum of *Mecp2*-null mice. Thus, decreased BDNF levels observed in RTT might be caused, on one hand, by direct MeCP2 regulation and, on the other hand, by miRNA-mediated repression [183]. Intriguingly, miR-132 regulates negatively MeCP2 levels and consequently reduces BDNF levels [201]. Furthermore, BDNF promotes miR-132 expression [202]. Taken together, these results suggest the existence of a homeostatic mechanism in the regulation of MeCP2 and BDNF expression through the involvement of miR-132. 

Few data are available so far concerning transcriptional alterations of long non-coding RNAs (lncRNA) in RTT. Esteller and coworkers [203] reported the first evidence of an aberrant expression (both up- and down-regulation) of lncRNAs in brain of *Mecp2*-null mice (Figure 4). In particular, the observed up-regulation of *AK081227* transcripts in *Mecp2*-null mice was associated with down-regulation of the nearby gene *gamma-aminobutyric acid receptor subunit Rho 2* (*Gabrr2*), encoding a factor involved in GABAergic signaling [204]. However, further studies are needed to clarify the role of these classes of ncRNAs in RTT pathogenesis.

Several studies pinpoint MeCP2 as a repressor of spurious transcription, through the silencing of transposons and repetitive elements [17,30,205,206] (Figure 4). The up-regulation of L1 transposons and intracisternal A particles has been reported in *Mecp2*-null cortical neurons [17]. Moreover, increased genomic amounts of L1 sequences have been reported in *Mecp2*-null neuroepithelial cells, *Mecp2*-null iPSC-derived neuronal precursor cells, and postmortem brain of RTT patients, which suggests an increased L1 retrotransposition [205]. In a more recent study, somatic insertion of human-specific L1 sequence (L1Hs) was found increased in brain of RTT patients [206]. Notably, uncontrolled L1 retrotransposition has been reported to cause insertion, deletion, or the formation of new splice-sites [207,208,209], with the subsequent effects on gene expression and cellular function. This allows one to speculate a pathogenetic role of aberrant retrotransposition in RTT.

Finally, an up-regulation of transcripts derived from both strands of pericentric heterochromatin (*MajSat-fw* and *MajSat-rv* transcripts) has been detected in nuclear extracts of *Mecp2*^−/y^ cortical neurons [17]. However, the analysis of expression of these ncRNAs by a strand-specific approach failed to reveal any mis-regulation of these transcripts in neurons derived from differentiation of murine embryonic stem cells [56]. Thus, the role of MeCP2 in the transcriptional regulation of this repetitive element is still debated.

## 6. Conclusions

Rett syndrome is a severe neurodevelopmental disorder that, although reversible in principle, nowadays remains largely incurable.

During recent decades, several research groups have attempted to decipher molecular mechanisms responsible for RTT pathogenesis, but this issue remains still unsolved to date. This is probably due to the fact that RTT arises from a combination of multiple dysfunctions rather than from the alteration of a single biological process.

At the molecular level, indeed, the absence of MeCP2 causes a global alteration of the neuronal epigenome, which includes the aberrant spreading of several histone modifications and the unbalanced distribution of molecular factors involved in the regulation of gene expression.

Furthermore, although the role of MeCP2 in the establishment/maintenance of DNA methylation is still debated, some large-scale studies have revealed several changes of different types of these epigenetic modifications in the RTT condition.

The epigenomic changes that occur in RTT parallel a general transcriptional deregulation in both directions, which concerns several classes of transcripts, such as protein-coding RNAs, miRNAs, and lncRNAs, thus leading to the impairment of diverse downstream pathways. Furthermore, several studies performed in RTT models have highlighted a higher expression of repetitive elements, the activation of which might have harmful effects on the genome.

Whether the transcriptional deregulation observed in RTT depends on changes in DNA methylation/hydroxymethylation or in the unbalanced distribution of epigenetic factors is still an open question, and, probably, these alterations arise from the synergistic combination of both phenomena.

The epigenomic and transcriptomic changes that occur in RTT strengthen the idea that, in physiological conditions, MeCP2 could act as a guardian of the neuronal epigenome by protecting methylated cytosines from oxidation, by limiting the spreading of histone acetylation and by restricting the uncontrolled binding of additional epigenetic factors, with the final result of ensuring a correct transcriptional program for neurons.

The deciphering of the impact of molecular factors that, upon MeCP2 dysfunction, have a detrimental effect on the stability of neuronal epigenome might be pivotal for the development of focused therapeutic strategies for RTT. Nowadays, indeed, it is well established that a large number of epigenetic readers and writers can be targeted by diverse epidrugs, thus allowing, potentially, for the correction of molecular defects associated with their abnormal functioning.
